# Assessment of the health status of middle-aged and elderly men with head scale, SF-36, IIEF5, AMS, and IPSS

**DOI:** 10.1186/s12877-021-02595-y

**Published:** 2021-11-12

**Authors:** Yi Zhu, Jian-Hui Li, Jing Zhao, Jun-Biao Zheng, Qun-Feng Liang, Xiao-Hua Yu, Shu-Cheng Zhang, Hui-Juan Shi, Wei-Jin Zhou, Qian-Xi Zhu

**Affiliations:** 1 Intensive Care Unit, The Fourth People’s Hospital of Zhenjiang, Zhenjiang, 212001 China; 2 Department of Reproductive Epidemiology and Social Science, NHC Key Lab of Reproduction Regulation, Shanghai Institute for Biomedical and Pharmaceutical Technologies, Fudan University, 779 Old Hu Min Road, Shanghai, 200237 China; 3 Department of Cardiology, the Key Laboratory of Cardiovascular Disease, The First Hospital of Lanzhou University, Lanzhou, 730000 China; 4 Department of Urology, The First People’s Hospital of Jiashan, Zhejiang, 314102 China; 5 Risk Adapted Prevention (RAD) Group, Division of Preventive Oncology, National Center for Tumor Diseases (NCT), German Cancer Research Center (DKFZ), 69120 Heidelberg, Germany; 6 Department of Cell Biology, Research Institute of National Health Commission of China, Beijing, 100081 China

**Keywords:** Aging, Aging males’ symptoms, Biological age, International index of erectile function, International prostate symptom score

## Abstract

**Background:**

Identifying practical and distinguished indicators and influencing factors of male aging may be useful in predicting subsequent aging trends, designing personalized prevention, and improving lifestyle and health.

**Methods:**

A cross-sectional, population-based study was performed in Jiashan County, China in 2016. A total of 690 local male residents, aged 40 to 80 years, were eligible for recruitment. Demographic and lifestyle information was collected through structured interviews. A self-designed head scale, the Medical Outcomes Study 36-item Short Form (SF-36), International Index of Erectile Function (IIEF5), Aging Males’ Symptoms (AMS), and International Prostate Symptom Score (IPSS) were used. Analysis of variance, local polynomial regression smoothing curves, multiple linear regression, and partial correlation analyses were performed.

**Results:**

All the scales deteriorated with increasing age (*P* < 0.01), especially from the age of 60. The most significant changes between adjacent age groups were found in IIEF5 scores (16.7, 43.5 and 39.4%). Income, nutrition, personality and neighborhood relationship had an effect on SF-36 and AMS after adjusting for age (*P* < 0.01). Furthermore, neighborhood relationship modified the age effect on the head scale score and IIEF5 (*P* = 0.03); nutrition modified the relationship between age and SF-36 (*P* < 0.01).

**Conclusions:**

Recession of reproductive health may be a distinct predictor of male aging. The associations of social inequalities or personality and health offer potential interventions for men’s health in aging. Self-reported scales may limit the precision and more physical fitness tests could be combined for a more precise assessment.

## Background

With an overall increase in life expectancy, quantifying the aging of different individuals has been brought into focus. The rates of biological aging are highly variable among individuals of the same chronological age. However, to date, there are no acknowledged aging markers to measure the biological age of an individual.

The epigenetic clock is thought to be the most promising molecular estimator of the biological age [1]. Some, including Horvath’s epigenetic clock, Hannum’s clock, and Levine’s clock, have been developed based on DNA methylation biomarkers for predicting aging for lifespan and healthspan [2–5]. Telomere length and epigenetic clock estimates are thought to be independent predictors of chronological age and mortality risk [6]. However, molecular biomarkers preclude their application to large-scale screens and routine physical examinations due to population specificity, invasiveness, and high costs.

In clinical settings, health-related quality of life (HRQoL) has been assessed using the Medical Outcomes Study 36-item Short Form (SF-36) questionnaire to evaluate the effect of disease or treatment [7, 8]. However, the application of SF-36 in the general population remains contradictory. An 18-year longitudinal study of civil servants (initially aged 35–55 years) based in London showed that physical health deteriorated while mental health tended to improve with age [9]. A general decline in the SF-36 score was found between 1995 and 2016 in the French population, but with wide disparities in trends between age groups [10]. A 2001–2011 US study found a declining SF-36 score in younger age groups, whereas older age groups tended to report higher mental scores [11].

The International Index of Erectile Function (IIEF5), Aging Males’ Symptoms (AMS) and International Prostate Symptom Score (IPSS) are commonly used scales for assessing the natural degradation of reproductive health in aging males [12, 13]. IIEF5, AMS and IPSS are used to assess erectile dysfunction, symptomatic late-onset hypogonadism, and lower urinary tract symptoms, respectively. The relationship among these scales has received attention [14, 15], but the relationship between reproductive health and overall health has seldom been studied. Recently, several studies in women indicated that reproductive health was a predictor of overall health. Irregular and long menstrual cycles in adolescence and adulthood were associated with a greater risk of premature mortality (age < 70 years), and these relationships were strongest for deaths related to cardiovascular disease [16]. Young women with a low number of harvested oocytes had an increased risk of aging-related events, such as cardiovascular diseases or osteoporosis, and early ovarian aging might serve as a marker of later accelerated aging [17]. For men and women, Sexual Health and Overall Wellness (SHOW) survey showed a strong association between satisfaction with sex and overall health [18]. However, relevant studies on men in order to elaborate the relationship or the predictive effect of reproductive health on overall health are still lacking.

Apart from health degradation, aging is accompanied by changes in appearance. Facial aging is considered the most prominent and readily accessible phenotype of human aging [19]. We attempted to assess aging using a self-designed scale of head conditions, including complexion, teeth, and hair.

Different aging rates among individuals indicate that aging-related changes are modifiable by environment, diet, lifestyle or variations in genetic background across different individuals. People from disadvantaged social classes age faster in terms of a quicker deterioration in physical health compared with people from advantaged social classes [9, 20]. The gap in health among people with high and low educational attainment increases with age [21]. It remains to explore the effect of other variables, such as nutrition and personality, on health decline.

Different clinical measures and physical signs may capture differences in lifespan and healthspan. To identify non-invasive and distinguished indicators of aging, we applied the five scales simultaneously, and appraised the trends with age, and correlations between them. Furthermore, we examined whether socioeconomic and other factors affected the trajectories of health decline.

## Methods

### Study population

The institutional review board of the Shanghai Institute for Biomedical and Pharmaceutical Technologies approved this research and its protocols. All methods were performed in accordance with the relevant guidelines and regulations. A cross-sectional, population-based survey was performed in Jiashan County, Zhejiang Province in 2016. A cluster sampling method was applied, and three villages were randomly selected. Local male residents, aged 40 to 80 years old, with no history of psychotic or cognitive disorders, or taking hormones or psychotropic agents, were noted as eligible for the recruitment.

### Clinical assessments

After obtaining written informed consent from volunteer participants, uniformly trained investigators collected demographic and lifestyle information through face-to-face structured interviews. In addition, five scales were administered to eligible participants to assess the different aspects of health status.

Income, nutrition, neighborhood relationship, and waist-to-hip ratio (WHR) were dichotomous variables: high and low, good and not good, close and alienated, and normal (< 0.9) and overweight (≥0.9), respectively. Personality was classified into three groups: extrovert, ambivert, and introvert. The body mass index (BMI) cut-off points were based on the guidelines of the Working Group on Obesity in China, defined as underweight (< 18.5 kg/m^2^), normal weight (18.5–23.9 kg/m^2^), and overweight (≥24 kg/m^2^). However, only 41 men were underweight, and they were classified into the normal weight group based on similar correlations with dependent variables.

The self-designed head scale contains five characteristics: complexion (poor, moderate, bright), lip color (pale, moderate, ruddy), teeth (loose, moderate, firm), hair quality (gray, a little gray, black and lustrous), and hair quantity (thinning, a little thinning, thick), for an overall score of 5 to 15 (higher score indicates better).

The Chinese version of the SF-36 questionnaire includes 36 items measuring eight health concepts: Physical Functioning, Role-Physical, Bodily Pain, General Health, Vitality, Social Functioning, Role-Emotional, and Mental Health. The precoded Likert-type item values were transformed into final item values [22]. To assess overall health status, we used the sum of all items of the eight health concepts, for an overall score of 0 to 145 (higher indicates better) [23].

The IIEF5 scale includes five questions, each scored from 1 to 5 (very low, low, moderate, high, very high, respectively), totaling 5 to 25 (higher indicates better) [24].

The AMS scale consists of 17 items, five for psychological, seven for somatic, and five for sexual subscores. Each item is scored from 1 to 5 (no, mild, moderate, severe, very severe, respectively), for an overall score of 17 to 85 (higher indicates severe) [25].

The IPSS includes seven questions, each scored from 0 to 5, totaling 0 to 35 (higher indicates severe) [26].

### Statistical analysis

The respondents were classified into four age groups by decade. Analysis of variance (ANOVA) was applied to compare the differences in the means of the five scales among the different age groups. Kernel-weighted local polynomial regression smoothing curves with confidence intervals were applied to explore the correlations between age and the five scale scores based on the different groups of demographic characteristics, including income, nutrition, personality, and neighborhood relationship. Based on the smoothing curves, we further fitted linear or fold-linear relationships to show the interactions between the variables. Finally, a multiple linear regression was constructed to analyse the possible influencing factors (Model I). Furthermore, two-way interaction terms between age and the above demographic characteristics were used to estimate whether age effects on scales were modified by these characteristics. If the interaction effect was significant (*P* < 0.05), then the interaction term was retained in the model (Model II). Pairwise correlations of the scales were analysed. To eliminate the influence of age, income, nutrition, personality, and neighborhood relationship, a partial correlation analysis was performed between the five scales.

All statistical analyses were performed using SAS 9.4 package (SAS Institute, Cary, NC, USA) and Stata 14 (Stata Corporation, College Station, TX, USA).

## Results

A total of 690 men, aged 60.7 ± 8.6 years old, with completed data were included in the statistical analysis. There were 103, 218, 271, and 98 men in 40–50 years, 51–60 years, 61–70 years, and 71–80 years age groups, respectively.

The results of the five scales differently degenerated with age. The head scale scores and IIEF5 decreased, and AMS increased with increasing age (for all four age groups); SF-36 began to decrease and IPSS began to increase significantly from the 61–70 years age group. When the adjacent age groups were compared, the most significant change percentages were found between the 51–60 years and 61–70 years age groups for all scales except IPSS. Taking IIEF5 as an example, the scores decreased by 16.7, 43.5, and 39.4%, respectively, between the adjacent age groups. Comparing the changes among the head scale, SF-36, IIEF5, AMS, and IPSS, the most significant changes were found in IIEF5 score. The change percentages of SF-36 were the smallest, only 1.5, 2.5, and 2.8%, respectively, with increasing age (Table 1).

**Table 1:** Means of health indexes in different age groups

Age	n	Headscore			SF-36			IIEF5			AMS			IPSS		
		Mean	SNK	Change%	Mean	SNK	Change%	Mean	SNK	Change%	Mean	SNK	Change%	Mean	SNK	Change%
**total**	690	9.6			128.8			14.2			27.4			10.8		
**40–50**	103	11.3	a	–	132.7	a	–	22.1	a	–	22.0	a	–	8.6	a	–
**51–60**	218	10.2	b	−9.7	130.7	a	−1.5	18.4	b	−16.7	25.0	b	13.6	9.6	a	11.6
**61–70**	271	9.0	c	−11.8	127.5	b	−2.5	10.4	c	−43.5	29.7	c	18.8	11.4	b	18.8
**71–80**	98	8.4	d	−6.7	123.9	c	−2.8	6.3	d	−39.4	33.1	d	11.4	14.0	c	22.8
**F**		67.8			13.2			113.0			46.5			17.4		
**P**		< 0.01			< 0.01			< 0.01			< 0.01			< 0.01		

If significant difference was found by ANOVA analysis, Student’s-Newman-Kuels (SNK) method was applied for multiple comparisons. Different letters (ie, a and b) mean there were significant difference between the two groups

Change% = (Mean_51_-Mean_40_)/Mean_40_×100% or (Mean_61_-Mean_51_)/Mean_51_×100% or (Mean_71_-Mean_61_)/Mean_61_×100%

**Fig. 1:**
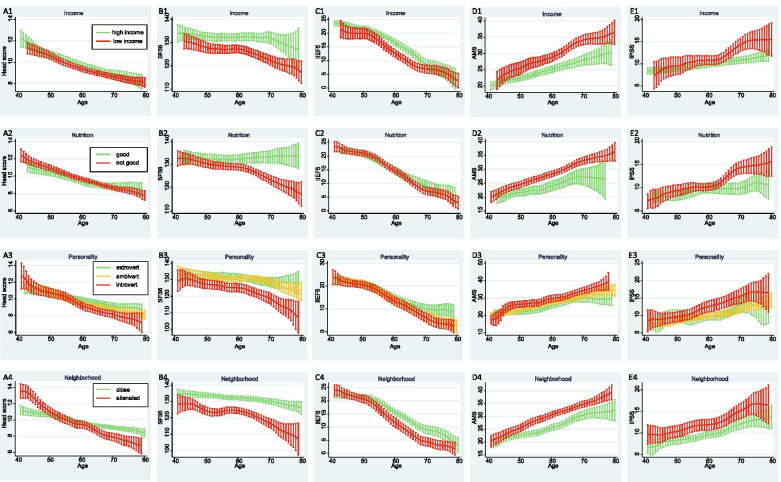
Kernel-weighted local polynomial regression smoothing curves of health indexes and age in different covariates groups. A) Sub-figures of correlations between head score and age, grouping according to income (A1), nutrition (A2), personality (A3) or neighborhood (A4). B) Sub-figures of correlations between SF-36 and age, grouping according to income (B1), nutrition (B2), personality (B3) or neighborhood (B4). C) Sub-figures of correlations between IIEF5 and age, grouping according to income (C1), nutrition (C2), personality (C3) or neighborhood (C4). D) Sub-figures of correlations between AMS and age, grouping according to income (D1), nutrition (D2), personality (D3) or neighborhood (D4). E) Sub-figures of correlations between IPSS and age, grouping according to income (E1), nutrition (E2), personality (E3) or neighborhood (E4)

**Fig. 2:**
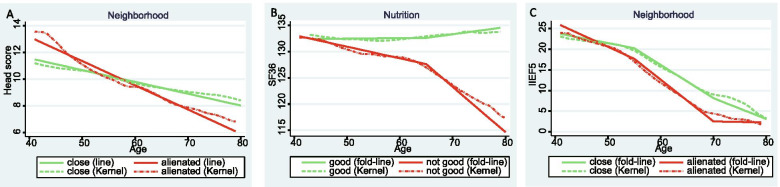
The line or fold-line regressions with interaction effect and corresponding Kernel-weighted local polynomial regression smoothing curves. **A** Sub-figure of regression on head score and age, grouping according to neighborhood relationship. **B** Sub-figure of regression on SF-36 and age, grouping according to nutrition. **C** Sub-figure of regression on IIEF5 and age, grouping according to neighborhood relationship

Kernel-weighted local polynomial smoothing curve, fold-linear fitting and multiple regression analyses showed that different covariates (income, nutrition, personality and neighborhood relationship) had different effects on the five scales, and some had modified effects on the relationship between age and the scales (Table 2, Figs. 1 and 2). The head scale score was slightly lower in the good-nutrition group. Neighborhood relationship modified the age effect on the head scale score and showed a slight X-shaped impact (Table 2 Model II, Fig. 1 A2, A4, and Fig. 2 A). Men with low income, introvert personality, and alienated neighborhood relationship had lower SF-36 scores. Nutrition showed a horizontal Y-shaped modification of the relationship between age and SF-36. SF-36 decreased rapidly after 65 years of age in the not good nutrition group (Table 2 Model II, Fig. 1 B1-B4 and Fig. 2 B). Men with a low income had lower IIEF5 scores. The neighborhood relationship showed first diverging and then converging modifications on the decreasing relationship between age and IIEF5, and IIEF5 decreased faster in men with alienated neighborhood relationship (Table 2 Model II, Fig. 1 C1, 1C4, and Fig. 2 C). Men with low income, not good nutrition, introvert personality, and alienated neighborhood relationship had higher AMS scores (Table 2 Model I, Fig. 1 D1-D4). Men with not good nutrition and alienated neighborhood relationship also had higher IPSS scores (Table 2 Model I, Fig. 1 E2 and E4). Other covariates, such as education, occupation, smoking, drinking, BMI, and WHR did not show an impact on the relationships between scale scores and age (figures and data are not shown).

**Table 2:** Health indexes in different socioeconomic status

Variable	n	Headscore			SF-36			IIEF5			AMS		IPSS	
		Mean	Model I ***k***(***P***)	Model II ***k***(***P***)	Mean	Model I ***k***(***P***)	Model II ***k***(***P***)	Mean	Model I ***k***(***P***)	Model II ***k***(***P***)	Mean	Model I ***k***(***P***)	Mean	Model I ***k***(***P***)
**Constant**	–	–	16.91 (< 0.01)	20.78 (< 0.01)	–	138.3 (< 0.01)	144.4 (< 0.01)	–	51.88 (< 0.01)	58.26 (< 0.01)	–	6.49 (< 0.01)	–	0.15 (0.93)
**Age**	690	–	−0.11 (< 0.01)	− 0.18 (< 0.01)	–	−0.25 (< 0.01)	−0.34 (< 0.01)	–	− 0.65 (< 0.01)	− 0.76 (< 0.01)	–	0.38 (< 0.01)	–	0.20 (< 0.01)
**Income**														
Low	333	9.39	–	–	125.8	–	–	12.48	–	–	28.78	–	11.32	–
High	357	9.61	0.22 (0.14)	0.21 (0.15)	128.9	3.26 (< 0.01)	3.13 (< 0.01)	14.66	2.14 (< 0.01)	2.11 (< 0.01)	26.34	−2.44 (< 0.01)	10.90	−0.46 (0.34)
**Nutrition**														
Not good	484	9.68	–	–	125.5	–	–	13.89	–	–	29.22	–	11.74	–
Good	206	9.33	−0.34 (0.02)	− 0.42 (< 0.01)	129.1	3.67 (< 0.01)	−15.62 (< 0.01)	13.24	− 0.68 (0.27)	− 0.81 (0.19)	25.90	−3.32 (< 0.01)	10.48	−1.30 (0.01)
**Age×Nutrition**	–	–	–	–	–	–	0.32 (< 0.01)	–	–	–	–	–	–	–
**Personality**														
Extrovert	250	9.72	–	–	128.6	–	–	14.20	–	–	26.43	–	10.88	–
Ambivert	284	9.53	−0.22 (0.02)	− 0.16 (0.07)	128.8	−1.75 (< 0.01)	− 1.76 (< 0.01)	13.02	− 0.45 (0.23)	− 0.35 (0.35)	27.46	1.17 (< 0.01)	10.34	0.47 (0.13)
Introvert	156	9.26			124.6			13.48			28.79		12.10	
**Neighborhood**														
Alienated	204	9.43	–	–	123.2	–	–	12.30	–	–	29.13	–	12.14	–
Close	486	9.58	0.15 (0.32)	−5.44 (< 0.01)	131.4	8.43 (< 0.01)	8.17 (< 0.01)	14.83	2.47 (< 0.01)	−6.80 (0.11)	25.98	−3.16 (< 0.01)	10.08	−2.20 (< 0.01)
**Age×Neighborhood**	–	–	–	0.09 (< 0.01)	–	–	–	–	–	0.15 (0.03)	–	–	–	–

Model I: multiple linear regression without interaction

Model II: multiple linear regression with interaction that existed significant effect (*P* < 0.05)

Correlation analysis showed that age, head scale score, SF-36, IIEF5, AMS and IPSS displayed pairwise correlations. The highest correlation was observed between IIEF5 and age (*r* = 0.63). Furthermore, when age, income, nutrition, personality, and neighborhood relationship were adjusted, the absolute values of correlation coefficients (partial r) decreased, and the significance of the correlation still existed, except between head scale score and IPSS or SF-36 and IIEF5 (Table 3).

**Table 3:** Pearson correlations and partial correlations between variables

Variable	Head score	SF-36	IIEF5	AMS	IPSS
**AGE**					
***r*(*P*)**	−0.51(< 0.01)	−0.24(< 0.01)	− 0.63(< 0.01)	0.46(< 0.01)	0.31(< 0.01)
**Head score**					
***r*(*P*)**		0.23(< 0.01)	0.44(< 0.01)	−0.33(< 0.01)	− 0.22(< 0.01)
***r***_**partial**_**(*P*)**^a^		0.11(< 0.01)	0.15(< 0.01)	−0.12(< 0.01)	− 0.07(0.08)
**SF-36**					
***r*(*P*)**			0.24(< 0.01)	−0.56(< 0.01)	− 0.29(< 0.01)
***r***_**partial**_**(*P*)**^a^			0.01(0.82)	−0.35(< 0.01)	− 0.16(< 0.01)
**IIEF5**					
***r*(*P*)**				−0.56(< 0.01)	− 0.29(< 0.01)
***r***_**partial**_**(*P*)**^a^				−0.35(< 0.01)	− 0.09(0.02)
**AMS**					
***r*(*P*)**					0.31(< 0.01)
***r***_**partial**_**(*P*)**^a^					0.14(< 0.01)

^a^Adjutsed for age, income, nutrition, personality and neighborhood relationship

## Discussion

All the indices, including the head scale scores, SF-36, IIEF5, AMS, and IPSS, deteriorated with increasing age, especially from the age of 60. IIEF5 showed the most significant degeneration; however, SF-36 showed the smallest degeneration. Income, nutrition, personality, and neighborhood relationship had effects and/or modified effects on health deterioration.

Various changes occur in human faces during aging. Chen utilized three-dimensional human facial morphologies for markers of aging [19]. Although examining the exterior facial morphological features is a non-invasive procedure and seems to be a reliable and convenient aging marker, the overall state of aging should not be restricted to the changes in soft tissues and skeleton structures on faces. We designed a scale to assess the aging characteristics of the head, including hair, facial skin, and teeth, and embodied the subjects’ self-assessment of appearance. As age increased, the head scale score decreased, and the group with alienated neighborhood relationship had a faster decrease. Counterintuitively, lower head scale scores were correlated with good nutrition, especially in the younger age groups. Nutrition is a time-varying variable that is easily changed by subjects’ status or conception. We speculate that appearance-conscious men were more likely to adjust their diet when they were unsatisfied with their appearance.

In our study, SF-36 worsened from the 61–70 years age group, but showed the smallest decrease compared to other indices. Ceiling effects (percent highest) were severe for Role physical, Bodily Pain, Social Functioning, and Role-Emotional of SF-36. SF-36 was generically applied to assess the effect of the presence of disease, treatment, or health promotion, and had differential validity [7, 8, 27]. However, studies in the general population have reported inconsistent trends in SF-36. A general decline in SF-36 scores was observed in repeated population-based cross-sectional surveys in France, and the largest decrease in score was observed among men aged 45–54 years in most dimensions of the SF-36 scale [10]. However, others found unexpectedly higher HRQoL, especially in mentally oriented scales, with increasing age in 18-year cohorts or cross-sectional studies [9, 11, 28]. Whether the SF-36 scale is suitable for the assessment of natural aging remains to be further studied, considering the discrimination of the scale and the cognitive ability of the population.

In addition, men with low income, introvert personality and alienated neighborhood relationship had lower SF-36 scores. Furthermore, the effect of nutrition diverged from 65 years of age, and not good nutrition increased vulnerability in old age. This finding is similar to that of Chandola et al., who reported that men from disadvantaged social classes might experience greater health declines with age [9]. In addition, Steptoe et al. highlighted that lower socioeconomic status (SES; wealth was used as the marker of SES in this article) is related to accelerated aging across a broad range of functional abilities and phenotypes, independent of the presence of health conditions, and that social circumstances impinge on multiple aspects of aging [20].

IIEF5, AMS and IPSS are commonly used scales for assessing male reproductive health. Most studies, including the Massachusetts Male Aging Study (MMAS) and the Men’s Attitudes to Life Events and Sexuality (MALES), reported the deterioration of the three aspects with increase in age in middle-aged and elderly men [14, 15, 29–31]. We identified some special features in our study. IIEF5 decreased with increased age more saliently than the other four indices, especially in the 61–70 years age groups. The longitudinal data confirmed that IIEF5 had a sharp turning period in the 60–70 years age range [13]. IPSS deteriorated later than IIEF5 and AMS.

The AMS score was susceptible to all four socioeconomic factors. Jankowska et al. also reported that age and education level constitute major determinants of the intensity of AMS [32]. There was a correlation between nutrition and IPSS, but none between nutrition and IIEF5. The results coincide with a study conducted in Southern China that reported that adequate fruit and vegetable intake, especially dark and leafy vegetables, were associated with IPSS among elderly Chinese men, but lacked an association with IIEF5 [33]. Interestingly, close or alienated neighborhood relationship showed a bifurcate impact on 55–75 year olds. This means that the interaction of neighborhood relationship and age increased the vulnerability of erectile function in older age. Plausible reasons for such an increase have been related to a long latency period of the effects of unhealthy experiences in earlier life, accumulation and interaction of economic and social capital throughout life, and increasing vulnerability in old age making differential exposure more harmful [9, 21].

Partial pairwise correlations between most outcomes still existed after adjusting for age. Vela-Navarrete et al. found that the relationship between IPSS and SF-36 was linear, with general increases in IPSS corresponding to impairment in quality of life, independent of age [31]. Aging on multisystem is concomitant, but the trigger mechanisms are still poorly understood.

Some limitations of this work should be acknowledged. Jiashan County is a representative county in East China. Subjects had poor awareness of self-care, and seldom exercised or sought medical treatment for health problems, except for serious diseases. The data were not significantly affected by treatment for reproductive health. Meanwhile, subjects might not have reported their health status precisely because of deficient health consciousness. That, to a certain extent, could explain the lack of discrimination of SF-36. Disease is related to scale scores, but the causal relationship is complicated. Disease may be an intermediate variable between socioeconomic factors and health status. Therefore, we did not include disease factors in the models to avoid overadjustment.

Analysis of personality types and relationship with neighbours provide new perspectives of the mental effects on aging. In our study, most assessment data were reported by subjects themselves. An individual’s perception of a decline health is recognized as a predictor of later poor health outcomes. In future studies, physical fitness tests could be combined for a more objective assessment, and follow-up data with subsequent morbidity will be needed.

Few studies take into account the trajectories of both overall health and reproductive health decline with increase in age, as well as the different effects of socioeconomic factors on health. Although the reproductive health of elderly men is still a neglected field, recession on the reproductive health of men may be an early predictor of aging. The results of our study revealed IIEF5, AMS, and a self-designed head scale as reliable, sensible, and convenient aging markers. Due to ceiling effects and indiscriminate traits with increasing age, the SF-36 scale still need to be assessed in the general population in China.

## Conclusions

Assessing the overall and reproductive health status may be useful to predict subsequent health trends, help track the aging process, design personalized medical treatment, and improve lifestyle and health. The associations between social inequalities or mental status and overall health or reproductive health offer potential avenues for facilitating remission and delay progression using nonpharmacological interventions. The benefits of such interventions for overall health in men may be far-reaching.

## Data Availability

The datasets during and/or analysed during the current study available from the corresponding author on reasonable request.

## Abbreviations

AMS: Aging Males’ Symptoms
ANOVA: Analysis of variance
BMI: The body mass index
HRQoL: Health-related quality of life
IIEF5: International Index of Erectile Function
IPSS: International Prostate Symptom Score
SES: socioeconomic status
SF-36: Medical Outcomes Study 36-item Short Form
SHOW: Sexual Health and Overall Wellness survey
WHR: Waist-to-hip ratio

## References

[1] Jylhävä J, Pedersen NL, Hägg S (2017). Biological age predictors. Ebiomedicine.

[2] Horvath S, Raj K (2018). DNA methylation-based biomarkers and the epigenetic clock theory of ageing. Nat Rev Genet.

[3] Horvath S. DNA methylation age of human tissues and cell types. Genome Biol. 2013;14:R115. 10.1186/gb-2013-14-10-r115 PMC4015143 24138928

[4] Hannum G, Guinney J, Zhao L, Zhang L, Hughes G, Sadda S (2013). Genome-wide methylation profiles reveal quantitative views of human aging rates. Mol Cell.

[5] Levine ME, Lu AT, Quach A, Chen BH, Assimes TL, Bandinelli S (2018). An epigenetic biomarker of aging for lifespan and healthspan. Aging (Albany NY).

[6] Marioni RE, Harris SE, Shah S, McRae AF, von Zglinicki T, Martin-Ruiz C (2018). The epigenetic clock and telomere length are independently associated with chronological age and mortality. Int J Epidemiol.

[7] Geijer M, Alenius GM, Andre L, Husmark T, Larsson PT, Lindqvist U (2020). Health-related quality of life in early psoriatic arthritis compared with early rheumatoid arthritis and a general population. Semin Arthritis Rheum.

[8] Forster-Horvath C, Unterreithmeier U, Fries S, Ganal S, Gutler J, Vogel N, et al. Mid term follow-up and assessment of cartilage thickness by arthro-MRI after arthroscopic cam resection, labral repair and rim trimming without labral detachment. Arthroscopy. 2021;37:541–51. 10.1016/j.arthro.2020.10.012 33359757

[9] Chandola T, Ferrie J, Sacker A, Marmot M (2007). Social inequalities in self reported health in early old age: follow-up of prospective cohort study. BMJ.

[10] Clause-Verdreau A, Audureau É, Leplège A, Coste J (2018). Contrasted trends in health-related quality of life across gender, age categories and work status in France, 1995–2016: repeated population-based cross-sectional surveys using the SF-36. J Epidemiol Commun H.

[11] Hanmer J, Kaplan RM (2016). Update to the report of nationally representative values for the noninstitutionalized US adult population for five health-related quality-of-life scores. Value Health.

[12] Corona G, Lee DM, Forti G, O'Connor DB, Maggi M, O'Neill TW (2010). Age-related changes in general and sexual health in middle-aged and older men: results from the European male ageing study (EMAS). J Sex Med.

[13] Zheng JB, Liang QF, Li JH, Zhang SC, Yu XH, Zhao J (2019). Longitudinal trends of AMS and IIEF-5 scores in randomly-selected community men 40 to 80 years old: preliminary results. J Sex Med.

[14] Atan A, Basar MM, Tuncel A, Mert C, Aslan Y (2007). Is there a relationship among age, international index of erectile function, international prostate symptom score, and aging males' symptoms score?. Int Urol Nephrol.

[15] Wong SY, Leung JC, Woo J (2009). A prospective study on the association between lower urinary tract symptoms (LUTS) and erectile dysfunction: results from a large study in elderly Chinese in southern China. J Sex Med.

[16] Wang Y, Arvizu M, Rich-Edwards JW, Stuart JJ, Manson JE, Missmer SA, et al. Menstrual cycle regularity and length across the reproductive lifespan and risk of premature mortality: prospective cohort study. BMJ. 2020;371:m3464. 10.1136/bmj.m3464 PMC7526082 32998909

[17] Christensen MW, Kesmodel US, Christensen K, Kirkegaard K, Ingerslev HJ (2020). Early ovarian ageing: is a low number of oocytes harvested in young women associated with an earlier and increased risk of age-related diseases?. Hum Reprod.

[18] Dean J, Shechter A, Vertkin A, Weiss P, Yaman O, Hodik M (2013). Sexual health and overall wellness (SHOW) survey in men and women in selected European and middle eastern countries. J Int Med Res.

[19] Chen W, Qian W, Wu G, Chen W, Xian B, Chen X (2015). Three-dimensional human facial morphologies as robust aging markers. Cell Res.

[20] Steptoe A, Zaninotto P (2020). Lower socioeconomic status and the acceleration of aging: an outcome-wide analysis. Proc Natl Acad Sci U S A.

[21] Ross CE, Wu CL (1996). Education, age, and the cumulative advantage in health. J Health Soc Behav.

[22] Ware JE, Snow KK, Kosinski M, Gandek B (1993). SF36 health survey manual and interpretation guide.

[23] Li L, Wang H, Shen Y (2002). Development and psychometric tests of a Chinese version of the SF-36 health survey scales. Zhonghua Yu Fang Yi Xue Za Zhi.

[24] Rosen RC, Cappelleri JC, Smith MD, Lipsky J, Pena BM (1999). Development and evaluation of an abridged, 5-item version of the international index of erectile function (IIEF-5) as a diagnostic tool for erectile dysfunction. Int J Impot Res.

[25] Heinemann LAJ, Zimmermann T, Vermeulen A, Thiel C, Hummel W (1999). A new 'aging males' symptoms' rating scale. Aging Male.

[26] Barry MJ, Fowler FJ, O'Leary MP, Bruskewitz RC, Holtgrewe HL, Mebust WK (1992). The American urological association symptom index for benign prostatic hyperplasia. The measurement Committee of the American Urological Association. J Urol.

[27] Alsubaie SF, Alkathiry AA, Abdelbasset WK, Nambi G (2020). The physical activity type Most related to cognitive function and quality of life. Biomed Res Int.

[28] Reile R, Helakorpi S, Klumbiene J, Tekkel M, Leinsalu M (2014). The recent economic recession and self-rated health in Estonia, Lithuania and Finland: a comparative cross-sectional study in 2004–2010. J Epidemiol Commun H.

[29] Travison TG, Shabsigh R, Araujo AB, Kupelian V, O'Donnell AB, McKinlay JB (2007). The natural progression and remission of erectile dysfunction: results from the Massachusetts male aging study. J Urol.

[30] Gonzalez-Sanchez B, Cendejas-Gomez J, Alejandro RJ, Herrera-Caceres JO, Olvera-Posada D, Villeda-Sandoval CI (2016). The correlation between lower urinary tract symptoms (LUTS) and erectile dysfunction (ED): results from a survey in males from Mexico City (MexiLUTS). World J Urol.

[31] Vela-Navarrete R, Alfaro V, Badiella LL, Fernandez-Hernando N (2000). Age-stratified analysis of I-PSS and QoL values in spanish patients with symptoms potentially related to BPH. Eur Urol.

[32] Jankowska EA, Szklarska A, Lopuszanska M, Medras M (2009). Age and social gradients in the intensity of aging males' symptoms in Poland. Aging Male.

[33] Liu ZM, Wong C, Chan D, Tse LA, Yip B, Wong SY (2016). Fruit and vegetable intake in relation to lower urinary tract symptoms and erectile dysfunction among southern Chinese elderly men: a 4-year prospective study of Mr OS Hong Kong. Medicine (Baltimore).

